# POLICY SERIES: PUBLIC HEALTH AND AGING POLICY: EXPERIENCE OF 2023 GSA SUMMER POLICY INTERNS

**DOI:** 10.1093/geroni/igad104.1235

**Published:** 2023-12-21

**Authors:** Patricia D’Antonio, Brian Lindberg

## Abstract

The Gerontological Society of America (GSA) is home to an established summer policy internship program. Aimed at emerging scholars, these professional development opportunities are named in memory of Kathryn Hyer, MPP, PhD, FGSA, FAGHE, and Greg O’Neill, PhD, who were policy scholars and long-time GSA members. Interns participate in an 8-week in person experience in Washington, DC. Interns will share how their experiences have impacted their research and their future career goals in gerontology.

